# Mobile App–Delivered Motivational Interviewing for Women on Eating Disorder Treatment Waitlists (MI-Coach: ED): Protocol for an App Development and Pilot Evaluation

**DOI:** 10.2196/66298

**Published:** 2025-04-10

**Authors:** Amané Halicki-Asakawa, Julia Mocci, Maya Libben

**Affiliations:** 1 Department of Psychology University of British Columbia Irving K. Barber Faculty of Arts and Social Sciences Kelowna, BC Canada

**Keywords:** eating disorders, motivational interviewing, treatment barriers, digital interventions, pilot test, protocol, eating disorder, eating, woman, women, female, Canada, Canadian, mobile apps, mobile health, mHealth, app development, app-based, mental health, pilot evaluation, waitlists, mixed methods, feasibility, acceptability, service delivery

## Abstract

**Background:**

A significant increase in eating disorder (ED) service waitlists has been observed in the past several years, exacerbating existing barriers to care (eg, long waitlists, scarcity of treatment centers, and positive beliefs surrounding pathology). Given that treatment delays have important clinical correlates (eg, entrenchment of ED pathology), exploring new methods of mental health service delivery for this population is of critical concern. App-based motivational interviewing (MI) delivered prior to the start of treatment has the potential to improve accessibility by simultaneously addressing structural (eg, travel costs) and individual (eg, low motivation) barriers to care. Despite the potential benefits, there remains a lack of empirically validated, ED-specific MI-based mobile apps. Evaluating the feasibility and acceptability of such interventions is a crucial first step before progressing to full-scale efficacy trials.

**Objective:**

This multiphasic mixed methods study aims to develop and assess the feasibility and acceptability of MI-Coach: ED, a novel app designed to increase motivation among women waitlisted for ED treatment. Specifically, this study seeks to determine participant engagement levels, user satisfaction, and perceived usability of the app, as well as to explore preliminary trends in motivation and ED-related symptoms following app use.

**Methods:**

Phase I adapted the content and interface of an existing app based on evidence-based principles (MI-Coach) for an ED population. Phase II pilot tested the app through a pre-post evaluation. Participants (n=30) aged 18 years and older were recruited from ED treatment waitlists in British Columbia, Canada. After completing baseline assessments evaluating demographic and clinical variables (eg, motivation, eating pathology, depression, and anxiety symptoms), participants were provided access to MI-Coach: ED for 1 month. Participants completed postintervention assessments and provided both quantitative and qualitative feedback on the app. Feasibility will be evaluated through the total number of participants recruited, study dropout rates, and engagement indicators (eg, modules completed) within the app. Acceptability will be assessed through self-report measures and semistructured exit interviews, which will explore user experiences, perceived benefits, and barriers to app engagement. Additionally, exploratory analyses will examine changes in motivation and ED symptoms before and after the intervention.

**Results:**

The MI-Coach: ED app has been developed, and recruitment was initiated in November 2022 and terminated in May 2024. Results are being analyzed and will be submitted for publication in May 2025.

**Conclusions:**

This study has the potential to transform ED service delivery and mitigate the impacts of existing treatment barriers for this population. By leveraging a digital MI-based intervention, MI-Coach: ED could serve as a scalable and accessible pretreatment tool, helping to bridge the gap between initial help-seeking and formal ED treatment. Findings from this study will inform the refinement of the intervention and recruitment strategies for future large-scale efficacy trials.

**International Registered Report Identifier (IRRID):**

DERR1-10.2196/66298

## Introduction

Eating disorders (EDs) are serious, life-threatening psychological illnesses that involve disturbances surrounding body image, emotion regulation, and eating behaviors [1,2], resulting in a number of deleterious health consequences and high levels of psychiatric comorbidity [3-5]. Despite the severity of these disorders, ED populations are significantly less likely to access mental health services compared to those with other disorders [5,6]. For instance, wait times exceeding 12 months for ED-specific services have been reported in Ontario, Canada, and nonprofit organizations have observed a 2-fold increase in requests for peer-support services following the COVID-19 pandemic [7]. In this regard, several treatment barriers have been identified limiting access to ED treatments and services. As EDs require specialized forms of care, treatment centers are typically few in number, necessitating significant travel time and associated costs (eg, costs related to travel and childcare and loss of income due to sick leave), and often resulting in long waitlist durations [6]. Further, ambivalence regarding treatment is common among ED populations, with motivation for recovery often in conflict with other factors, such as engaging in ED behaviors to cope with difficult emotions or to provide a sense of control [8-10].

Unfortunately, treatment delays are associated with adverse outcomes, such as reductions in pretreatment levels of motivation and poorer long-term prognosis. Early intervention has been associated with improved treatment outcomes following discharge, longer durations between relapses, and improvements in related psychological symptoms such as depression and anxiety [11]. Recent efforts to bridge treatment barriers and improve outcomes for this population include the delivery of brief interventions such as motivational interviewing (MI) prior to the start of formal treatment. Initially developed by Miller and Rollnick [12] to address ambivalence within substance use treatment, MI is a person-centered approach that aims to strengthen the individual’s intrinsic motivation to change [13]. A recent systematic review conducted by Denison-Day et al [8] found substantial support for the use of MI interventions for individuals with EDs, with a majority (74%) of the studies reviewed reporting significant and long-term improvements in motivation as a result of MI interventions compared to passive interventions, such as psychoeducation. Broadly, studies report that brief MI interventions lead to improvements in motivation, self-esteem, depressive symptoms, and positive treatment outcomes (eg, greater engagement in treatment), particularly when delivered prior to the start of formal treatment [14-16].

Despite the potential for pretreatment MI interventions to address individual treatment barriers, they are typically conducted through in-person, face-to-face sessions [8,14,16,17]. In-person interventions may not adequately address structural treatment barriers, such as costs and scarcity of treatment providers. In order to address this issue, internet and communications technology (ICT)–based interventions have been proposed as a low-cost and accessible form of delivering ED interventions [5,18-20]. Apps in particular show potential in the delivery of pretreatment MI interventions, as they are easy to use, require low effort, and become habitual over time. Further, unlike other ICT-based interventions, they have high hedonic motivation (ie, they are enjoyable to use), which increases their use and adoption [21,22]. In addition, the digital components embedded within apps (eg, modules, graphic design, and diverse multimedia content) have been shown to significantly improve adherence to interventions and reduce dropout rates [23].

Despite preliminary findings suggesting that apps may be an acceptable means of delivering ED services, there is a paucity of effective, evidence-based apps available for use [24-27]. Most publicly available app interventions are based on CBT and behavioral self-monitoring principles but demonstrate limited efficacy compared to traditional delivery methods [24,28]. However, considering the accessibility of apps and their wide adoption in various health and social contexts [29], they may be an effective mode of delivery for pretreatment MI contexts. Help-seeking individuals waiting for formal services likely do not have access to in-person treatment options, despite their urgent need for services. Further, as they have not yet started formal ED treatment, receiving a pretreatment MI intervention may significantly improve treatment outcomes. For example, such interventions may increase motivation to stay on the waitlist and adhere to treatment once services are received [30]. An app-based MI intervention thus has the potential to simultaneously address structural and individual treatment barriers. Despite the potential benefits, to date, no research has investigated the use of app-based MI pretreatment interventions for EDs.

The proposed study aims to adapt and pilot-test MI-Coach, a widely available app-based MI platform, for individuals on ED treatment waitlists. Created by Resiliens Inc, MI-Coach is an evidence-based self-help app developed in collaboration with a registered clinical psychologist. The app was adapted for use within an ED pretreatment context. Furthermore, this will be the first study to investigate whether an app-based MI pretreatment intervention improves clinical symptoms, motivation for treatment, and openness to change for individuals while they wait for ED treatment.

## Methods

### Hypotheses

This project is guided by the technology acceptance model (TAM), which theorizes that an ICT’s acceptability is determined by its perceived ease of use and usefulness [31]. It is thus hypothesized that greater perceived usefulness and ease of use of the MI-Coach: ED app will lead to greater use and engagement with the app, and subsequently, improved treatment outcomes. It is also hypothesized that participants will experience an improvement in their motivation to recover from an ED following use of the app. In addition, the app’s acceptability and feasibility will be examined, as well as the possible impacts of other clinical variables (eg, depression, anxiety, BMI, and ED severity) on motivation to recover.

### Ethical Considerations

Ethics approvals were granted by the University of British Columbia Okanagan Campus’ Behavioral Research Ethics Board (H22-02046) on October 4, 2022, and by the Vancouver Coastal Health Research Institute (V22-02046) on October 10, 2023. All eligible participants provided informed consent electronically through the secure web-based platform Qualtrics prior to enrollment, after receiving detailed information about study procedures, potential risks and benefits, data confidentiality, and voluntary participation. Participants were informed of their right to withdraw from the study at any time without penalty. Participant privacy was maintained by storing data on password-protected university servers accessible only to research personnel. All identifying information was stored separately from the study data. Participants received compensation of up to CAD $40 (≈US $28.41) in the form of digital gift cards. Compensation was clearly outlined in the informed consent form and was structured to avoid influencing participation or app use.

### Study Design

The current pilot test evaluation follows a naturalistic pre-post design. A pilot test framework was chosen to assess the initial feasibility and acceptability of the app and to identify any potential issues with the study design and delivery format prior to evaluating the app’s efficacy at a larger scale [32]. The MI-Coach: ED app was designed to improve motivation in women-identifying people waiting for formal ED treatment and will be pilot-tested in the province of British Columbia, Canada. Qualitative and descriptive data were collected to determine the acceptability and feasibility of the MI-Coach: ED app.

### Study Population

#### Sample Size Estimation

Given the focus of this study on the acceptability and feasibility, rather than the efficacy, of the MI-Coach: ED app, formal analytic methods were not used to estimate the prehoc sample size. In keeping with previous feasibility studies in the ED field [14,33,34] and pilot study guidelines, which suggest that modest sample sizes based on the pragmatics of recruitment are sufficient when evaluating the processes, feasibility, and preliminary acceptability of novel interventions [32,35], a sample size of 30 participants was selected, recruited while they wait for ED services and treatments in British Columbia, Canada. Additionally, it was determined that qualitative interviews would be conducted with interested participants on a rolling basis until thematic saturation is reached [36].

#### Participant Recruitment

ED treatment providers and clinicians located in British Columbia were contacted by email and asked to distribute study recruitment flyers providing an overview of the study, incentives, and instructions to contact the study team to indicate their interest to clients on their waitlists (Multimedia Appendix 1). To address existing barriers to ED treatment (eg, logistical challenges and clinician burnout [6,37]) and research [38], a multipronged approach to recruitment has been used in previous studies [15,39], whereby clinician-based referrals are supplemented by web-based recruitment and partnerships with peer support organizations. As such, clinician-based referrals were supplemented by web-based recruitment strategies, including postings on the National Eating Disorder Information Center website and social media, as well as partnerships with peer support organizations. Additionally, a research partnership was established with a local provincial health authority, the Vancouver Coastal Health Eating Disorders Program, which embedded the study flyer as part of an initial resource package provided to patients newly added to their waitlist. Given the findings that recruitment strategies influence sample characteristics and engagement [38], this multipronged approach aimed to maximize the accessibility of the study and diversity within the study sample.

#### Eligibility Criteria

Eligibility criteria were (1) being on a waitlist to receive services for an ED-specific concern and not for a separate mental disorder in British Columbia, (2) being at least 18 years of age, (3) self-identifying as a woman, (4) fluency in written and spoken English, (5) meeting the criteria for a diagnosis of a threshold or subthreshold ED (ie, BN, AN-R, AN-BP, BED, and OSFED), (6) absence of psychosis or diagnosis of a schizophrenia-spectrum disorder, and (7) absence of cognitive impairments or sensory deficits that may interfere with technology use (eg, hearing impairments and recent traumatic brain injury).

### Intervention

MI-Coach is a self-help app created by Resiliens Inc [40] targeting motivation to engage in health behavior change (eg, exercising, smoking cessation, and sleep hygiene), and is widely available on both Apple and Android app stores. Developed in collaboration with a clinical psychologist and MI expert from the University of Queensland (Stan Steindl, PhD), the app features eight modules, consisting of over 35 lessons led by a registered clinical psychologist with accompanying exercises. In addition, the app includes short articles, mood and behavior assessments, mindfulness-based audio tracks, summary screens and analytics, community-based discussion groups, and the ability to communicate directly with a trained mental health professional.

For the purposes of this study, the content of MI-Coach was tailored for an ED population. In line with best practices for developing ED-specific digital interventions [17,30,41], an in-depth review of potentially iatrogenic or triggering content in the existing app was identified. Specifically, content referring to weight loss or health-focused exercise goals was identified, which prior research suggests may inadvertently reinforce disordered eating behaviors [28,30,37,42]. Subsequently, a literature review was conducted to identify MI topics and ED behavior change goals used in previous interventions, such as exploring reasons for and against pursuing treatment, validating the difficulties of addressing ED pathology, and identifying values and goals driving ED recovery efforts [11,17,30,41].

**Figure 1:**
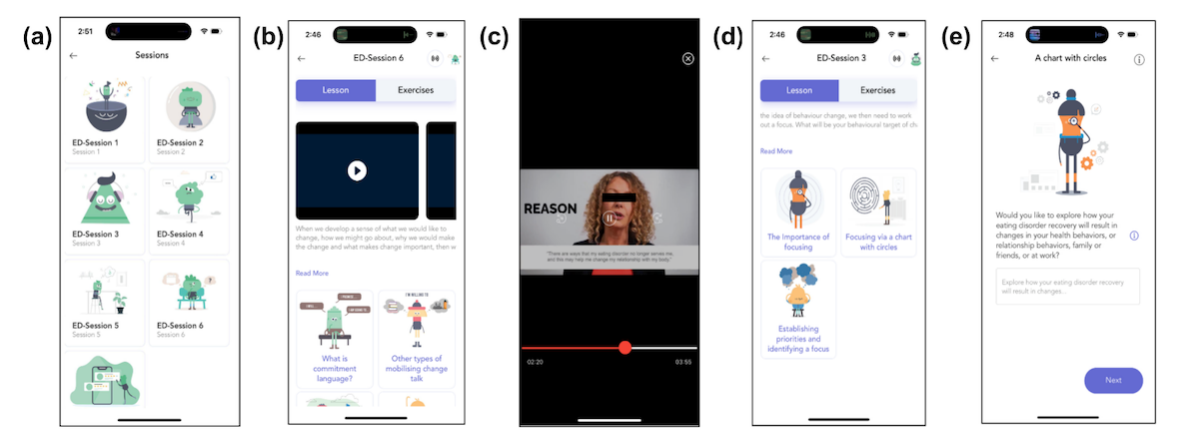
Screenshots of the MI-Coach: ED interface illustrating (a) the home screen with session modules, (b) example module content, (c) brief videos, (d) examples of exercises available per module, and (e) example exercise content. Each feature targets ED-specific barriers such as ambivalence or low treatment motivation. ED: eating disorder.

Following approaches outlined in previous research [15], the research team then performed iterative content analyses to remove or reframe any references misaligned with ED recovery principles (eg, shifting from weight-centric to motivation-centered goals that explore the individual’s reasons for seeking treatment, and the inclusion of topics such as ambivalence and identification of goals and values relevant to recovery). The overall structure and MI principles used in the original app were retained given their demonstrated effectiveness within an ED context [8]. Throughout this process, informal stakeholder consultations were conducted with ED-specialized clinicians involved in the project, who verified the appropriateness of the revised app content. All video lessons were subsequently refilmed using the University of British Columbia Okanagan’s recording studio and equipment. The new videos featured a clinical psychologist with ED treatment expertise to reduce discrepancies between the original and adapted app content (eg, clinical expertise and years of training). Examples of the MI-Coach: ED interface are provided in Figure 1.

Once the adaptation of app content was completed, a new section was built into the interface of the existing MI-Coach app by technological partners (Resiliens Inc) to house the adapted content. Several components of the base app (ie, assessments, audio tracks, analytics, discussion groups, and communication with the mental health professional) were removed to reduce the scope of the project and to protect participant confidentiality. The final adapted MI-Coach: ED app consisted of seven modules (Multimedia Appendix 2) targeting motivation to engage in ED recovery behaviors (eg, help-seeking, values exploration, and self-acceptance) covering the following topics sequentially: ambivalence and behavior change, self-acceptance and compassionate motivation, exploring possible behavior change goals, sustain and change talk, commitment language, and relapse prevention.

### Outcomes and Measures

The primary outcomes that will be evaluated are the overall acceptability and feasibility of the MI-Coach: ED (Multimedia Appendix 3)*.* Feasibility will be assessed through attrition rates throughout the study period, frequency of app use, and module completion. Acceptability will be assessed through qualitative and quantitative feedback from participants through semistructured interviews and self-reports. Additionally, exploratory analyses will be conducted regarding the impacts of the MI-Coach: ED app on motivation and associated clinical characteristics.

Self-reported demographic data, including gender identity, age, level of education, ethnicity, and socioeconomic status were collected using Qualtrics, a web-based and secure data collection platform. Additionally, clinical data (ie, weight and height, history of ED treatment, age of ED onset, presence of cognitive difficulties, sensory deficits, history of traumatic brain injury, other mental health comorbidities, and ED waitlist experiences) was collected. The Sick, Control, One, Fat, and Food questionnaire [43], a brief screener that identifies the presence of common ED symptoms, was used to determine eligibility prior to enrollment. Clinical characteristics (ie, eating pathology, recovery motivation, body dissatisfaction, depression, and anxiety symptoms) were evaluated through several self-report questionnaires administered at baseline and posttest, including the Eating Disorder Examination Questionnaire (EDE-Q) [44], the Readiness and Motivation for Change Questionnaire (RMQ) [45], the Body Shape Questionnaire (BSQ) [46], the Patient Health Questionnaire-9 (PHQ-9) [47], and the 7-item General Anxiety Disorder Scale (GAD-7) [48]. Additionally, technological literacy was evaluated at baseline using the Mobile Device Proficiency Questionnaire [49] and the eHealth Literacy Scale [50].

Quantitative measures of feasibility and acceptability of the MI-Coach: ED app were assessed using an adapted version of the TAM Questionnaire [31] and the user version of the Mobile App Rating Scale (uMARS) [51]. The adapted version of the TAM questionnaire includes changes to each item to better fit the research question and sample characteristics of this study (eg, “Using CHART-MASTER in my job would enable me to accomplish tasks more quickly” was modified to “Using MI-Coach: ED allowed me to access mental health services more quickly”). An optional semistructured interview was conducted with interested participants to assess their experiences with the app and to further evaluate the feasibility and acceptability of MI-Coach: ED.

**Figure 2:**
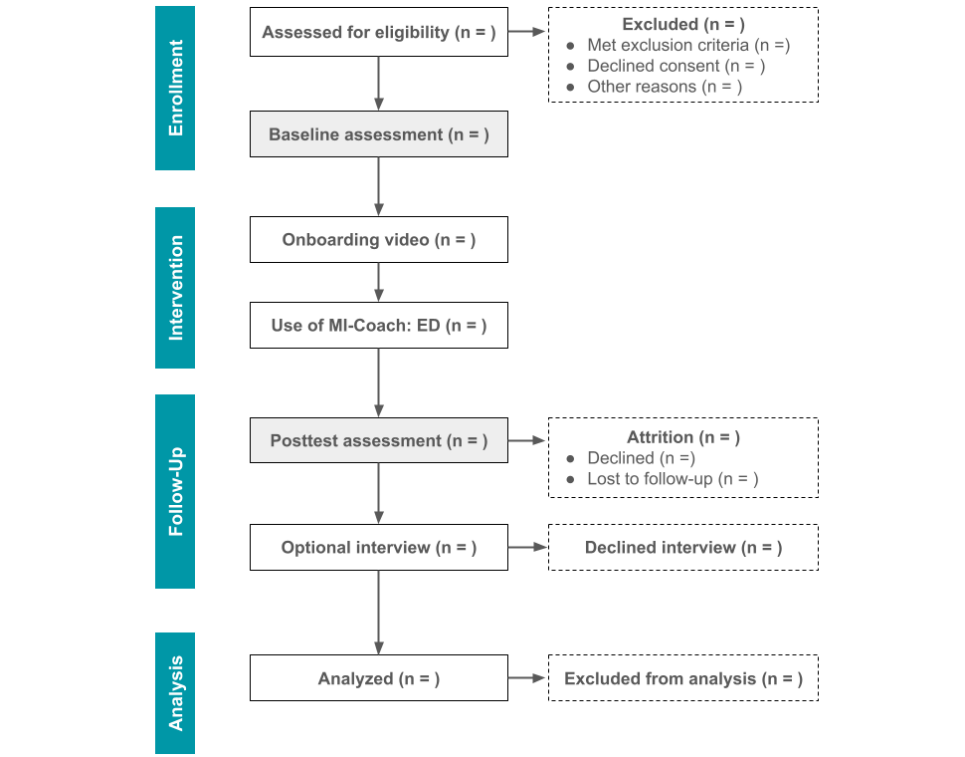
Participant flow diagram.

An overview of participant flow is provided in Figure 2. Participants were recruited between November 2022 and April 2024. Clinicians and administrators working in a range of ED treatment settings (eg, provincial health authorities, nonprofit charities, and private practice clinics) were contacted via email and asked to share the recruitment advertisement with clients on their waitlists. Interested participants were asked to communicate with the study team via email to enroll in the study. Once initial contact was established, a 15-minute telephone screener was conducted by the study team to assess participants’ eligibility for the study and to provide additional details regarding the study. The Sick, Control, One, Fat, and Food questionnaire was used to evaluate the presence of ED symptoms. Once eligibility was established, consent (Multimedia Appendix 4) and baseline measures were collected through Qualtrics, including a demographics questionnaire, the EDE-Q, the RMQ, the PHQ-9, the GAD-7, and the BSQ. Following this, an onboarding session was conducted through a secure videoconferencing platform (ie, Zoom), during which participants were given a demonstration on downloading and using the app and provided their log-in information.

The subsequent pilot-test portion of the study consisted of a four-week period during which participants used the app in a naturalistic way. Following study completion, participants were compensated CAD $10 (≈US $7.10) for each study portion completed (ie, baseline questionnaire package, onboarding session, posttest questionnaire package, and exit interview), receiving up to CAD $40 (≈US $28.41) through the Tango Card rewards platform. To reduce the impact of possible confounds (eg, financial motivation), participants were informed that their compensation would not be impacted by their app use during the pilot test phase and that there are no minimum requirements for app use. Once the pilot test was completed, a second brief consent form (Multimedia Appendix 5) and posttest questionnaires assessing self-reported clinical features (ie, the EDE-Q, RMQ, PHQ-9, GAD-7, and BSQ) and feedback regarding the app (ie, the adapted TAM-Q and uMARS) were completed. Finally, participants were invited to participate in a videoconferencing-based exit interview to provide additional feedback on their experience with the study author. The semistructured interview guide (Multimedia Appendix 6) was used as an outline to standardize the interview process across participants [52].

### Statistical Analysis

Prior to analysis, interview audio recordings will be transcribed verbatim by 2 trained research assistants and verified by the primary researcher (AH-A). Qualitative interview transcripts will be analyzed using the NVivo software package (version 12; QSR International), to assess in-depth feedback regarding MI-Coach: ED’s feasibility and acceptability. A thematic content analysis will be conducted to analyze the qualitative responses from the open-ended uMARS questionnaire and semistructured interview data using Braun and Clarke’s [36] framework. A bottom-up theoretical approach was used, in which the analysis was driven by the data generated through the semistructured interviews [36,53].

Additionally, quantitative analyses will be conducted using the R statistical package (version 4.2.0; R Core Team). Descriptive analyses of posttest feedback questionnaires, attrition data, and sessions completed will be used to determine the feasibility and acceptability of the MI-Coach: ED app. Means, SDs, ranges, and percentages will be calculated for all descriptive variables (ie, age, ethnicity, BMI, height, and weight), and total and mean scores will be calculated for app acceptability measures. Overall app use (ie, number of modules completed, days that the app was used, assessments and exercises completed, and logins) will be calculated using in-app data provided by Resiliens Inc.

Finally, tests of clinical significance [54] will be used to evaluate changes in clinical presentation across time points. The following categorizations will be used to identify whether change is clinically significant: reliably improved, in which there is reliable change and the clinical cutoff is no longer met; reliably deteriorated, in which there is a reliable change in the disordered direction and the individual’s score falls within the clinical range; and unchanged, in which neither criteria of the Jacobson-Truax method are met [54,55]. Finally, exploratory growth curve analyses will be used to model individual participants’ change trajectories across the 4-week pilot test period [56].

## Results

The MI-Coach: ED app has been developed and is available for download and use. Recruitment was initiated in November 2022 and terminated in May 2024. Data analysis is currently underway, and we anticipate reporting results through peer-reviewed academic publications in May 2025.

## Discussion

### Anticipated Results

This study aimed to evaluate the feasibility and acceptability of MI-Coach: ED, an app designed to support motivation for ED recovery among individuals on treatment waitlists. It is anticipated that the intervention will be feasible to implement, with a high rate of participant engagement with the app, and that MI-Coach: ED would be perceived as an acceptable and useful tool for supporting motivation during the waitlist period. Prior research suggests that digital mental health interventions targeting ED populations are generally acceptable and feasible to users, particularly when they incorporate evidence-based therapeutic principles such as MI [15,28]. Additionally, app-based self-help interventions have demonstrated high engagement rates, which MI-Coach: ED was designed to include [23,27]. Furthermore, we anticipate that exploratory analyses will demonstrate preliminary improvements in motivation and ED-related symptoms following app use, given findings that brief MI-based interventions can enhance motivation for change, improve treatment engagement, and reduce ambivalence in ED populations [8,14,16,17]. As motivation is a key predictor of ED treatment outcomes [11], even small gains during the waitlist period could have meaningful clinical implications.

### Conclusions

Individuals with EDs face substantial barriers to accessing appropriate care, despite their urgent need for treatment [37,57,58]. Though both MI and app-based interventions have been suggested as ways to support treatment-seeking ED patients, in isolation, these interventions may not adequately address treatment barriers. For example, in-person MI interventions may be inaccessible to individuals in rural and remote locations, and interventions currently delivered through apps have limited evidence to support their efficacy [24]. As such, this study aims to simultaneously address individual and structural treatment barriers by developing and pilot-testing an MI-based app intervention for women on waitlists for ED treatment.

Providing app-based interventions to individuals who are waiting for ED treatment has the potential to improve treatment outcomes and represents a largely barrier-free, positive-approach method of addressing the growing problem of wait times for mental health care. Although this pilot study focuses on a modest sample, existing intervention frameworks and ED-focused digital interventions demonstrate how pilot studies can be used to guide the adaptation and evaluation of novel interventions through large-scale clinical trials [15,20,28]. For example, collecting and implementing end user feedback prior to formal efficacy testing has been suggested to ensure that the final intervention is used by the targeted population [28]. Furthermore, exploratory analyses on motivation and ED-related symptoms will help identify meaningful clinical outcome measures and establish preliminary effect size estimates to guide the design and power calculations for a full-scale randomized controlled trial. Additionally, by collecting demographic and digital literacy data, this study will provide insights into the app’s accessibility across diverse populations and inform potential adaptations to increase inclusivity. Future evaluations will also aim to follow participants into formal psychological treatment to assess the long-term clinical impact of pretreatment MI interventions.

Given that prior research has demonstrated that multimodal recruitment modalities can enhance sample diversity, improve engagement, and ensure the inclusion of individuals with varying levels of symptom severity in digital intervention studies [38], data collected through this pilot study will be particularly valuable in refining future participant engagement strategies. In this regard, feasibility data collected from this study (including recruitment and retention rates, participant engagement with the app, and qualitative user feedback) will directly inform refinements to MI-Coach: ED and recruitment methods for subsequent large-scale efficacy evaluations, ensuring that the intervention is optimally structured for broader implementation. For example, future studies may continue to use a multipronged approach, such as the use of social media campaigns to supplement clinician referrals [38], and partnerships with provincial authorities may be expanded to integrate study recruitment directly into clinical workflows given their success in prior research [59]. As such, this pilot study serves as a crucial step in optimizing MI-Coach: ED for large-scale implementation and has the potential to provide tangible psychological support to its participants while contributing to the reconceptualization of ED service delivery.

## Appendix

Multimedia Appendix 1 Study recruitment flyer.

Multimedia Appendix 2 Overview of MI-Coach: ED Modules.

Multimedia Appendix 3 Study measures and outcome variables.

Multimedia Appendix 4 Consent form.

Multimedia Appendix 5 Brief consent form.

Multimedia Appendix 6 Qualitative interview guide.

## Abbreviations

BSQ: Body Shape Questionnaire
ED: eating disorder
EDE-Q: Eating Disorder Examination Questionnaire
GAD-7: 7-item General Anxiety Disorder Scale
ICT: internet and communications technology
MI: motivational interviewing
PHQ-9: Patient Health Questionnaire-9
RMQ: Readiness and Motivation for Change Questionnaire
TAM: Technology Acceptance Model
uMARS: user version of the Mobile App Rating Scale

## References

[1] (2022). Diagnostic and Statistical Manual of Mental Disorders.

[2] Klump KL, Bulik CM, Kaye WH, Treasure J, Tyson E (2009). Academy for eating disorders position paper: eating disorders are serious mental illnesses. Int J Eat Disord.

[3] Ágh T, Kovács G, Supina D, Pawaskar M, Herman BK, Vokó Z, Sheehan DV (2016). A systematic review of the health-related quality of life and economic burdens of anorexia nervosa, bulimia nervosa, and binge eating disorder. Eat Weight Disord.

[4] Mitchison D, Hay P, Slewa-Younan S, Mond J (2012). Time trends in population prevalence of eating disorder behaviors and their relationship to quality of life. PLoS One.

[5] Weissman RS, Rosselli F (2017). Reducing the burden of suffering from eating disorders: unmet treatment needs, cost of illness, and the quest for cost-effectiveness. Behav Res Ther.

[6] Innes NT, Clough BA, Casey LM (2017). Assessing treatment barriers in eating disorders: a systematic review. Eat Disord.

[7] Health Quality Ontario (2023). Eating DIsorders: Care for People of All Ages.

[8] Denison-Day J, Appleton KM, Newell C, Muir S (2018). Improving motivation to change amongst individuals with eating disorders: a systematic review. Int J Eat Disord.

[9] Schoen EG, Lee S, Skow C, Greenberg ST, Bell AS, Wiese JE, Martens JK (2012). A retrospective look at the internal help-seeking process in young women with eating disorders. Eat Disord.

[10] Williams S, Reid M (2010). Understanding the experience of ambivalence in anorexia nervosa: the maintainer's perspective. Psychol Health.

[11] Clausen L, Lübeck M, Jones A (2013). Motivation to change in the eating disorders: a systematic review. Int J Eat Disord.

[12] Miller W, Rollnick S (1991). Motivational Interviewing: Preparing to Change Addictive Behaviour.

[13] Miller WR, Rollnick S (2012). Motivational Interviewing: Helping People Change.

[14] Feld R, Woodside DB, Kaplan AS, Olmsted MP, Carter JC (2001). Pretreatment motivational enhancement therapy for eating disorders: a pilot study. Int J Eat Disord.

[15] Muir S, Newell C, Griffiths J, Walker K, Hooper H, Thomas S, Thomas PW, Arcelus J, Day J, Appleton KM (2017). MotivATE: a pretreatment web-based program to improve attendance at UK outpatient services among adults with eating disorders. JMIR Res Protoc.

[16] Weiss CV, Mills JS, Westra HA, Carter JC (2013). A preliminary study of motivational interviewing as a prelude to intensive treatment for an eating disorder. J Eat Disord.

[17] Geller J, Dunn EC (2011). Integrating motivational interviewing and cognitive behavioral therapy in the treatment of eating disorders: tailoring interventions to patient readiness for change. Cognit Behav Pract.

[18] Bauer S, Moessner M (2013). Harnessing the power of technology for the treatment and prevention of eating disorders. Int J Eat Disord.

[19] Cooper M, Reilly EE, Siegel JA, Coniglio K, Sadeh-Sharvit S, Pisetsky EM, Anderson LM (2022). Eating disorders during the COVID-19 pandemic and quarantine: an overview of risks and recommendations for treatment and early intervention. Eat Disord.

[20] Shingleton RM, Richards LK, Thompson-Brenner H (2013). Using technology within the treatment of eating disorders: a clinical practice review. Psychotherapy (Chic).

[21] Chandrashekar P (2018). Do mental health mobile apps work: evidence and recommendations for designing high-efficacy mental health mobile apps. Mhealth.

[22] Yuan S, Ma W, Kanthawala S, Peng W (2015). Keep using my health apps: discover users' perception of health and fitness apps with the UTAUT2 model. Telemed E-Health.

[23] Linardon J, Shatte A, Tepper H, Fuller-Tyszkiewicz M (2020). A survey study of attitudes toward, and preferences for, e-therapy interventions for eating disorder psychopathology. Int J Eat Disord.

[24] Juarascio AS, Manasse SM, Goldstein SP, Forman EM, Butryn ML (2015). Review of smartphone applications for the treatment of eating disorders. Eur Eat Disord Rev.

[25] Kim JP, Sadeh-Sharvit S, Darcy AM, Neri E, Vierhile M, Robinson A, Tregarthen J, Lock JD (2018). The utility and acceptability of a self-help smartphone application for eating disorder behaviors. J Technol Behav Sci.

[26] Sadeh-Sharvit S, Kim JP, Darcy AM, Neri E, Vierhile M, Robinson A, Tregarthen J, Lock JD (2018). Subgrouping the users of a specialized app for eating disorders. Eat Disord.

[27] Tregarthen J, Paik Kim J, Sadeh-Sharvit S, Neri E, Welch H, Lock J (2019). Comparing a tailored self-help mobile app with a standard self-monitoring app for the treatment of eating disorder symptoms: randomized controlled trial. JMIR Ment Health.

[28] Linardon J, Shatte A, Messer M, Firth J, Fuller-Tyszkiewicz M (2020). E-mental health interventions for the treatment and prevention of eating disorders: an updated systematic review and meta-analysis. J Consult Clin Psychol.

[29] Lee M, Lee H, Kim Y, Kim J, Cho M, Jang J, Jang H (2018). Mobile app-based health promotion programs: a systematic review of the literature. Int J Environ Res Public Health.

[30] Fursland A, Erceg-Hurn DM, Byrne SM, McEvoy PM (2018). A single session assessment and psychoeducational intervention for eating disorders: impact on treatment waitlists and eating disorder symptoms. Int J Eat Disord.

[31] Davis FD (1989). Perceived usefulness, perceived ease of use, and user acceptance of information technology. MIS Q.

[32] Leon AC, Davis LL, Kraemer HC (2011). The role and interpretation of pilot studies in clinical research. J Psychiatr Res.

[33] Rom S, Miskovic-Wheatley J, Barakat S, Aouad P, Fuller-Tyszkiewicz M, Maguire S (2022). Evaluating the feasibility and potential efficacy of a brief eTherapy for binge-eating disorder: a pilot study. Int J Eat Disord.

[34] Völker U, Jacobi C, Barr Taylor C (2011). Adaptation and evaluation of an internet-based prevention program for eating disorders in a sample of women with subclinical eating disorder symptoms: a pilot study. Eat Weight Disord.

[35] Bowen DJ, Kreuter M, Spring B, Cofta-Woerpel L, Linnan L, Weiner D, Bakken S, Kaplan CP, Squiers L, Fabrizio C, Fernandez M (2009). How we design feasibility studies. Am J Prev Med.

[36] Braun V, Clarke V (2006). Using thematic analysis in psychology. Qual Res Psychol.

[37] Ali K, Farrer L, Fassnacht DB, Gulliver A, Bauer S, Griffiths KM (2017). Perceived barriers and facilitators towards help-seeking for eating disorders: a systematic review. Int J Eat Disord.

[38] Vollert B, von Bloh P, Eiterich N, Beintner I, Hütter K, Taylor CB, Jacobi C (2020). Recruiting participants to an internet-based eating disorder prevention trial: impact of the recruitment strategy on symptom severity and program utilization. Int J Eat Disord.

[39] Leung SF, Ma J, Russell J (2012). Breaking the silence of eating disorders with the hope of an online self-help programme. Contemp Nurse.

[40] Resiliens Inc MI coach: a comprehensive app for finding your motivation for change. Resiliens.

[41] Hötzel K, von Brachel R, Schmidt U, Rieger E, Kosfelder J, Hechler T, Schulte D, Vocks S (2014). An internet-based program to enhance motivation to change in females with symptoms of an eating disorder: a randomized controlled trial. Psychol Med.

[42] Pretorius N, Arcelus J, Beecham J, Dawson H, Doherty F, Eisler I, Gallagher C, Gowers S, Isaacs G, Johnson-Sabine E, Jones A, Newell C, Morris J, Richards L, Ringwood S, Rowlands L, Simic M, Treasure J, Waller G, Williams C, Yi I, Yoshioka M, Schmidt U (2009). Cognitive-behavioural therapy for adolescents with bulimic symptomatology: the acceptability and effectiveness of internet-based delivery. Behav Res Ther.

[43] Morgan JF, Reid F, Lacey JH (1999). The SCOFF questionnaire: assessment of a new screening tool for eating disorders. BMJ.

[44] Fairburn CG, Beglin SJ (1994). Assessment of eating disorders: interview or self-report questionnaire?. Int J Eat Disord.

[45] Geller J, Brown KE, Srikameswaran S, Piper W, Dunn EC (2013). The psychometric properties of the readiness and motivation questionnaire: a symptom-specific measure of readiness for change in the eating disorders. Psychol Assess.

[46] Cooper PJ, Taylor MJ, Cooper Z, Fairbum CG (1987). The development and validation of the body shape questionnaire. Int J Eat Disord.

[47] Kroenke K, Spitzer RL, Williams JB (2001). The PHQ-9: validity of a brief depression severity measure. J Gen Intern Med.

[48] Spitzer RL, Kroenke K, Williams JBW, Löwe B (2006). A brief measure for assessing generalized anxiety disorder: the GAD-7. Arch Intern Med.

[49] Roque NA, Boot WR (2018). A new tool for assessing mobile device proficiency in older adults: the mobile device proficiency questionnaire. J Appl Gerontol.

[50] Norman CD, Skinner HA (2006). eHEALS: the eHealth literacy scale. J Med Internet Res.

[51] Stoyanov SR, Hides L, Kavanagh DJ, Wilson H (2016). Development and validation of the user version of the mobile application rating scale (uMARS). JMIR Mhealth Uhealth.

[52] Kallio H, Pietilä AM, Johnson M, Kangasniemi M (2016). Systematic methodological review: developing a framework for a qualitative semi-structured interview guide. J Adv Nurs.

[53] Moltu C, Stefansen J, Svisdahl M, Veseth M (2012). Negotiating the coresearcher mandate—service users' experiences of doing collaborative research on mental health. Disabil Rehabil.

[54] Jacobson N, Truax P (1992). Clinical significance: a statistical approach to defining meaningful change in psychotherapy research. Methodological Issues & Strategies in Clinical Research.

[55] Lambert M, Bailey R (2012). Measures of clinically significant change. APA Handbook of Research Methods in Psychology.

[56] Mirman D (2017). Growth Curve Analysisvisualization Using R.

[57] Arcelus J, Mitchell AJ, Wales J, Nielsen S (2011). Mortality rates in patients with anorexia nervosa and other eating disorders. A meta-analysis of 36 studies. Arch Gen Psychiatry.

[58] Treasure J, Schmidt U, Van FE (2003). Handbook of Eating Disorders.

[59] Brownstone L, Anderson K, Beenhakker J, Lock J, Le Grange D (2012). Recruitment and retention in an adolescent anorexia nervosa treatment trial. Int J Eat Disord.

