# Supplementary figures and images for: Mobile App–Delivered Motivational Interviewing for Women on Eating Disorder Treatment Waitlists (MI-Coach: ED): Protocol for an App Development and Pilot Evaluation
Source: JMIR Res Protoc. 2025 Apr 10;14:e66298. doi: 10.2196/66298 (PMC12022520; doi:10.2196/66298)

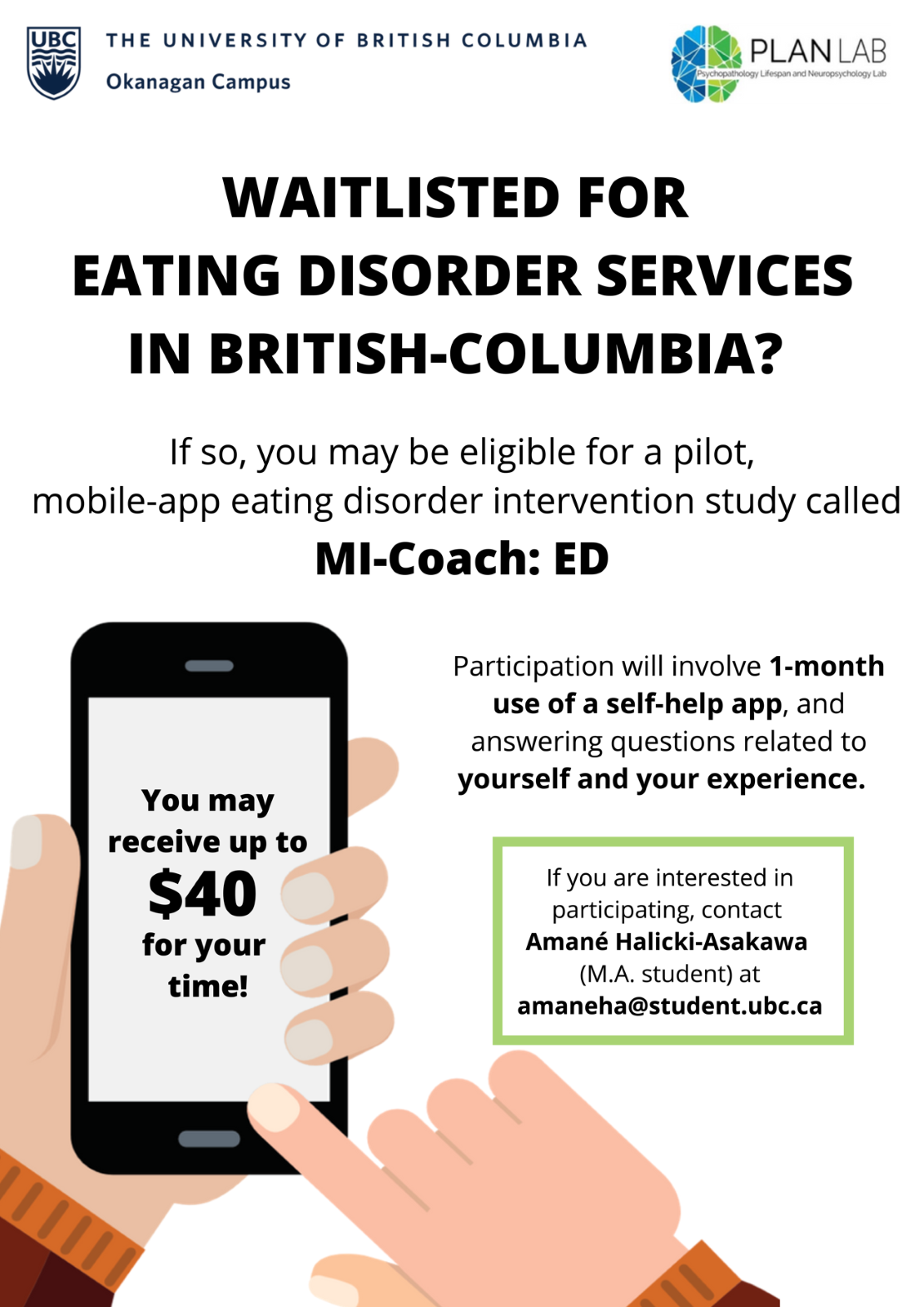

Supplement: Multimedia Appendix 1 [file resprot_v14i1e66298_app1.docx]
