# Supplementary material for: Mobile App–Delivered Motivational Interviewing for Women on Eating Disorder Treatment Waitlists (MI-Coach: ED): Protocol for an App Development and Pilot Evaluation
Source: JMIR Res Protoc. 2025 Apr 10;14:e66298. doi: 10.2196/66298 (PMC12022520; doi:10.2196/66298)
Supplement: Multimedia Appendix 2 [file resprot_v14i1e66298_app2.docx]

| Session | Content | Exercise(s) included in session |
| --- | --- | --- |
| Session 1 – Introduction | Understanding Behaviour change, the challenges one faces when considering change, and the role ambivalence plays.  Introduction to Motivational Interviewing (MI) to work through challenges, explore and resolve ambivalence, and find the motivation to engage in behaviour change | 1. Self-Reflection Exercise 2. Exploring Ambivalence: Physical Exercise 3. Exploring Ambivalence: Importance and Confidence |
| Session 2 – Creating Safeness | Begin building a particular type of relationship with ourselves that helps us to feel safe.  This includes being our own partner in health, being self-accepting and self-respecting, and cultivating a compassionate motivation towards ourselves.  In Motivational Interviewing all of this is referred to as the MI Spirit. | 1. Calming the Mind and Body 2. Accepting and Respecting Yourself |
| Session 3 – Let’s Focus | Focusing on specific areas of change through a structured process, culminating a focus while remaining open to revising priorities and focus | 1. Preparing for a Chart with Circles 2. A Chart with Circles |
| Session 4 – Exploring (Part 1) | Introduction to the concept of sustain talk and change talk. | 1. Responding to Dr. Bossy with “Yes but..” 2. What is your sustain talk? 3. Exploring Arguments for Change |
| Session 5 – Exploring (Part 2) | Dive deeper into change talk, elaborating on all the many and varied arguments for change. | 1. Exploring Your Change Talk |
| Session 6 – Commitment and Planning | The concept of commitment language and how such commitments are predictive of actual change. | 1. Always Bring It Back to the Body 2. Exploring confidence 3. Getting started 4. Making change fun and rewarding 5. Reducing procrastination 6. Remembering to act 7. Confidence statements 8. Affirming personal strengths and qualities 9. Learning from the success of others 10. Always bring it back to the body - Exploring Importance 11. Exploring Discrepancies 12. What will you change? 13. Refining the Change goal 14. Developing the plan 15. Aligning with Values 16. Valued function replacement |
| Session 7 – Reflect and Review | Troubleshooting the common challenges and how to manage relapses. | 1. Reviewing MI Coach: ED 2. How did you go? 3. Troubleshooting Challenges 4. Celebrating Successes 5. Lapse vs. Relapse 6. Defining Risk Situations 7. Risk and Leave Management Plan |
