# Supplementary material for: Mobile App–Delivered Motivational Interviewing for Women on Eating Disorder Treatment Waitlists (MI-Coach: ED): Protocol for an App Development and Pilot Evaluation
Source: JMIR Res Protoc. 2025 Apr 10;14:e66298. doi: 10.2196/66298 (PMC12022520; doi:10.2196/66298)
Supplement: Multimedia Appendix 3 [file resprot_v14i1e66298_app3.docx]

| **Outcomes** | **Measures** | **Response Options** | **Baseline (week 0)** | **Post-test (week 4)** | **Source** |
| --- | --- | --- | --- | --- | --- |
| ***General characteristics*** | In what country were you born? | DESCRIPTIVE TEXT | X |  | Original |
|  | What is your current age? | DESCRIPTIVE TEXT |  |  |  |
|  | What is your gender identity? [Select one] | 1. Cisgender man 2. Cisgender woman 3. Transgendered man 4. Transgendered woman 5. Nonbinary person 6. Prefer not to answer 7. Other (please specify) |  |  |  |
|  | What are your preferred pronouns? | DESCRIPTIVE TEXT |  |  |  |
|  | How would you describe your ethnic or racial background? [Select all that apply] | 1. Black/African American (e.g., African, Afro-Caribbean, African-Canadian, etc.) 2. East Asian (e.g., Chinese, Korean, Japanese, Taiwanese, etc.) 3. Latinx (e.g., Latin American, Hispanic, etc.) 4. Middle Eastern (e.g., Arab, Persian, Afghan, Egyptian, Iranian, etc.) 5. South Asian (e.g., East Indian, Pakistani, Sri Lankan, Indo-Caribbean, etc.) 6. Southeast Asian (e.g., Filipino, Vietnamese, Cambodian, Thai, etc.) 7. White (e.g., European descent) 8. Indigenous (e.g., First Nations, Inuit, Métis, etc.) 9. Oceania origins (e.g., Australian, New Zealander, Pacific Islander, etc.) 10. Prefer not to answer 11. Other (please specify) |  |  |  |
|  | What is your highest degree or level of school completed? [Select one] | 1. Less than grade 12 2. High school graduate, diploma or equivalent 3. Trades/technical/vocational training 4. Some university/college credit, no degree 5. Bachelor's degree 6. Graduate degree |  |  |  |
|  | What was your total household income before taxes last year? [Select one] | 1. 0 - $29,999 2. $30,000-$49,999 3. $50,000-$69,999 4. $70,000-$99,999 5. $100,000-$149,999 6. $150,000 or more 7. Do not know 8. Prefer not to answer |  |  |  |
|  | Have you ever been diagnosed with any other mental disorder by a health care professional (e.g., psychologist, psychiatrist, counsellor, nurse, social worker, family doctor, etc.) | 1. Yes [Specify – DESCRIPTIVE TEXT] 2. No |  |  |  |
|  | Have you ever been diagnosed with a schizophrenia-spectrum disorder (e.g., Schizophrenia, Schizotypal personality disorder), or experienced an episode of psychosis? | 1. Yes 2. No |  |  |  |
|  | Have you experienced a traumatic brain injury (e.g., concussion) or other cognitive symptoms (e.g., stroke, loss of hearing or vision) in the past year? | 1. Yes 2. No |  |  |  |
|  | Do you have any other physical health conditions? | 1. Yes [Specify – DESCRIPTIVE TEXT] 2. No |  |  |  |
| ***Eating disorder treatment history*** | What is your current height? [Specify if feet/inches or meters/centimeters] | DESCRIPTIVE TEXT | X |  | Original |
|  | What is your current weight? [Specify if pounds or kilograms] | DESCRIPTIVE TEXT |  |  |  |
|  | Have you ever been diagnosed with an eating disorder by a health care professional (e.g., psychologist, psychiatrist, counsellor, nurse, social worker, family doctor, etc.) | 1. Yes 2. No |  |  |  |
|  | Around what age did you first start experiencing eating disorder symptoms? | DESCRIPTIVE TEXT |  |  |  |
|  | Have you ever been treated for an eating disorder? | 1. Yes 2. No |  |  |  |
|  | If yes: when was the last time that you received treatment? | DESCRIPTIVE TEXT |  |  |  |
|  | If yes: in what type of treatment facility did you receive services? [Please select all that apply] | 1. Peer support services (i.e., seeing someone who is trained to provide support, but is not a health care professional, on a regular basis) 2. Outpatient treatment (i.e., seeing a mental health professional, such as a psychologist, counsellor, or social worker, on a regular basis at a private practice, clinic, or hospital setting) 3. Day treatment (i.e., staying at a hospital during the day for most days of the week) 4. Inpatient treatment (i.e., staying at a hospital during the day and overnight, for most days of the week) 5. Residential treatment (i.e., receiving overnight treatment at a setting other than a hospital, such as a private residence or clinic) 6. Other (Specify) |  |  |  |
|  | If yes: please describe any other times that you received treatment for your eating disorder | DESCRIPTIVE TEXT |  |  |  |
|  | Are you currently on a waitlist for eating disorder treatment? | 1. Yes 2. No |  |  |  |
|  | If yes: how long have you been waiting for treatment? [Specify if days/weeks/months/years] | DESCRIPTIVE TEXT |  |  |  |
|  | If yes: which of the following treatment centre waitlists are you currently on? [Select all that apply] | 1. Government-funded programs [select from the following list]:    1. Abbotsford Eating Disorders Services (Abbotsford, BC)    2. Adolescent Outpatient Services at St. Joseph’s Hospital (Comox, BC)    3. Adult Mental Health Substance Use Team in Nanaimo (Nanaimo, BC)    4. BC Children’s Kelty Mental Health Resource Centre (Vancouver, BC)    5. Campbell River North Island Eating Disorders Program (Campbell River, BC)    6. Central Island Child and Youth Eating Disorders Program (Nanaimo, BC)    7. Child & Youth Mental Health Eating Disorders Program (Duncan, BC)    8. Chilliwack Mental Health – Eating Disorders (Chilliwack, BC)    9. Comox Valley North Island Eating Disorders Program (Comox, BC)    10. Discovery Vista House (Vancouver, BC)    11. East Kootenay Eating Disorders Program (Cranbook, BC)    12. Eating Disorders Program – South Vancouver Island Region (Victoria, BC)    13. Eating Disorders Program run by NARSF (Nanaimo, BC)    14. Fraser South Eating Disorders Program (Delta, BC)    15. Jessie’s Legacy Eating Disorders Prevention and Awareness Program (North Vancouver, BC)    16. Kamloops Eating Disorders Program (Kamloops, BC)    17. Kelowna Eating Disorders Program (Kelowna, BC)    18. Kootenay Boundary Eating Disorder Clinic (Castlegar, BC)    19. Lions Gate Hospital Medical Stabilization (North Vancouver, BC)    20. North Fraser Eating Disorders Program (Port Moody, BC)    21. North Okanagan Eating Disorders Program (Vernon, BC)    22. Northern Regional Eating Disorders Clinic (Prince George, BC)    23. Outpatient Dietitian Services for Central Island (Nanaimo, BC)    24. Port Alberni Mental Health and Substance Use (Port Alberni, BC)    25. Provincial Specialized Eating Disorders Program for Adults (Vancouver, BC)    26. Richmond Eating Disorders Program (Richmond, BC)    27. TeleEating Disorders, Island Health (Victoria, BC)    28. The Looking Glass Foundation for Eating Disorders (Vancouver, BC)    29. The Looking Glass Residence (Vancouver, BC)    30. Vancouver Coastal Health Eating Disorders Program (Vancouver, BC)    31. Victoria General Hospital (Victoria, BC)    32. Williams Lake Eating Disorders Program (Williams Lake, BC) 2. Private practice/clinician(s) [Specify names] 3. Other [Specify] |  |  |  |
|  | If yes: please describe any additional information pertaining to your time waiting for eating disorder treatment | DESCRIPTIVE TEXT |  |  |  |
| ***Current eating disorder symptomatology*** | On how many of the past 28 days…   - - - - Have you gone for long periods of time (8 waking hours or more) without eating anything at all in order to influence your shape or weight       - Have you tried to exclude from your diet any foods that you like in order to influence your shape or weight (whether or not you have succeeded)?       - Have you tried to follow definite rules regarding your eating (for example, a calorie limit) in order to influence your shape or weight (whether or not you have succeeded)?       - Have you had a definite desire to have an empty stomach with the aim of influencing your shape or weight?       - Have you had a definite desire to have a totally flat stomach?       - Has thinking about food, eating, or calories made it very difficult to concentrate on things that you are interested in (for example, working, following a conversation, or reading)?       - Has thinking about shape or weight made it very difficult to concentrate on things you are interested in (for example, working, following a conversation, or reading)?       - Have you had a definite fear of losing control over eating?       - Have you had a definite fear that you might gain weight?       - Have you felt fat?       - Have you had a strong desire to lose weight? | 0 = no days  1 = 1 – 5 days  2 = 6 – 12 days  3 = 13 – 15 days  4 = 16 – 22  5 = 23 – 27  6 = Everyday | X | X | Adapted from Fairburn & Beglin, 1994 |
|  | - - - - Over the past 28 days, how many times have you eaten what other people would regard as an unusually large amount of food (given the circumstances)?       - On how many of these times did you have a sense of having lost control over your eating (at the time that you were eating)?       - Over the past 28 days, on how many DAYS have such episodes of overeating occurred (i.e., you have eaten an usually large amount of food and have had a sense of loss of control at the time)?       - Over the past 28 days, how many times have you made yourself sick (vomit) as a means of controlling your shape or weight?       - Over the past 28 days, how many times have you taken laxatives as a means of controlling your shape or weight)?       - Over the past 28 days, how many times have you exercised in a “driven” or “compulsive” way as a means of controlling your weight, shape or amount of fat, or to burn off calories? | DESCRIPTIVE TEXT |  |  |  |
|  | - - - - Over the past 28 days, on how many days have you eaten in secret (i.e., furtively)? …. Do not count episodes of binge eating.       - On what proportion of the times that you have eaten have you felt guilty (felt that you’ve done wrong) because of its effect on your shape or weight? …. Do not count episodes of binge eating       - Over the past 28 days, how concerned have you been about other people seeing you eat? …. Do not count episodes of binge eating | 0 = no days  1 = 1 – 5 days  2 = 6 – 12 days  3 = 13 – 15 days  4 = 16 – 22  5 = 23 – 27  6 = Everyday |  |  |  |
|  | - - - - Has your weight influenced how you think about (judge) yourself as a person?       - Has your shape influenced how you think about (judge) yourself as a person?       - How much would it have upset you if you had been asked to weigh yourself once a week (no more, or less, often) for the next four weeks?       - How dissatisfied have you been with your weight?       - How dissatisfied have you been with your shape?       - How uncomfortable have you felt seeing your body (for example, seeing your shape in the mirror, in a shop window reflection, while undressing or taking a bath or shower)?       - How uncomfortable have you felt about others seeing your shape or figure (for example, in communal changing rooms, when swimming, or wearing tight clothes)? | 0 = not at all  1 – 2 = Slightly  3 – 4 = Moderately  5 – 6 = Markedly |  |  |  |
| ***Readiness and motivation to engage in treatment*** | - In the past two weeks, how much of you has wanted to restrict your eating? (please circle one of the following percentages) - In the past two weeks, how much of you has been actively working to eat more? (please circle one of the following percentages) | 0 – 30% = A small part of me  31 – 60% = About half of me  61 – 100% = Most of me | X | X | Adapted from Geller et al., 2013 |
|  | - If you were to reduce your restriction (i.e. Eat more), how much of this would be for you (versus for others)? (please circle one of the following percentages) | 0 – 30% = Not very much for me  31 – 60% = About half for me  61 – 100% = Mostly for me |  |  |  |
|  | - If you decided to reduce your restriction (i.e., eat more), how confident are you in your ability to do so? (please circle one of the following percentages) | 0 – 30% = Not at all confident  31 – 70 % = Confident  61 – 100% = Extremely confident |  |  |  |
|  | - In the past two weeks, how much of you has wanted to maintain a low weight (please circle one of the following percentages) - In the past two weeks, how much of you has been actively working to gain weight? (please circle one of the following percentages) | 0 – 30% = A small part of me  31 – 60% = About half of me  61 – 100% = Most of me |  |  |  |
|  | - If you were to gain weight, how much of this would be for you (versus for others)? (please circle one of the following percentages) | 0 – 30% = Not very much for me  31 – 60% = About half for me  61 – 100% = Mostly for me |  |  |  |
|  | - If you decided to gain weight, how confident are you in your ability to do so? (please circle one of the following percentages) | 0 – 30% = Not at all confident  31 – 70 % = Confident  61 – 100% = Extremely confident |  |  |  |
|  | - In the past two weeks, how much of you has wanted to lose weight? (please circle one of the following) - In the past two weeks, how much of you has been actively working to stop your efforts to lose weight? (please circle one of the following percentages) | 0 – 30% = A small part of me  31 – 60% = About half of me  61 – 100% = Most of me |  |  |  |
|  | - If you were to stop your efforts to lose weight how much of this would be for you (versus for others)? (please circle one of the following percentages) | 0 – 30% = Not very much for me  31 – 60% = About half for me  61 – 100% = Mostly for me |  |  |  |
|  | - If you decided to stop your efforts to lose weight, how confident are you in your ability to do so? (please circle one of the following percentages) | 0 – 30% = Not at all confident  31 – 70 % = Confident  61 – 100% = Extremely confident |  |  |  |
|  | - In the past two weeks, how much of you has not wanted to have periods? (please circle one of the following) - In the past two weeks, how much of you has been actively working to get your periods back? (please circle one of the following) | 0 – 30% = A small part of me  31 – 60% = About half of me  61 – 100% = Most of me |  |  |  |
|  | - If you were to try to get your periods back, how much of this would be for you (versus for others)? (please circle one of the following) | 0 – 30% = Not very much for me  31 – 60% = About half for me  61 – 100% = Mostly for me |  |  |  |
|  | - If you decided to try to get your periods back, how confident are you in your ability to do so? (please circle one of the following) | 0 – 30% = Not at all confident  31 – 70 % = Confident  61 – 100% = Extremely confident |  |  |  |
|  | - In the past two weeks, how much of you has wanted to binge? (please circle one of the following) - In the past two weeks, how much of you has been actively working to reduce your bingeing? (please circle one of the following) | 0 – 30% = A small part of me  31 – 60% = About half of me  61 – 100% = Most of me |  |  |  |
|  | - If you were to reduce your bingeing, how much of this would be for you (versus for others)? (please circle one of the following) | 0 – 30% = Not very much for me  31 – 60% = About half for me  61 – 100% = Mostly for me |  |  |  |
|  | - If you decided to reduce your bingeing, how confident are you in your ability to do so? (please circle one of the following) | 0 – 30% = Not at all confident  31 – 70 % = Confident  61 – 100% = Extremely confident |  |  |  |
|  | - In the past two weeks, how much of you has wanted to restrict to compensate for binges? (please circle one of the following) - In the past two weeks, how much of you has been actively working to reduce your restriction before or after a binge? (please circle one of the following) | 0 – 30% = A small part of me  31 – 60% = About half of me  61 – 100% = Most of me |  |  |  |
|  | - If you were to reduce your restriction before or after a binge, how much of this would be for you vs. Others? (please circle one of the following) | 0 – 30% = Not very much for me  31 – 60% = About half for me  61 – 100% = Mostly for me |  |  |  |
|  | - If you decided to reduce your restriction before or after a binge, how confident are you in your ability to do so? (please circle one of the following) | 0 – 30% = Not at all confident  31 – 70 % = Confident  61 – 100% = Extremely confident |  |  |  |
|  | - In the past two weeks, how much of you has wanted to make yourself sick? (please circle one of the following) - In the past two weeks, how much of you has been actively working to reduce the amount you make yourself sick? (please circle one of the following) | 0 – 30% = A small part of me  31 – 60% = About half of me  61 – 100% = Most of me |  |  |  |
|  | - If you were to reduce the amount you make yourself sick, how much of this would be for you (versus for others)? (please circle one of the following) | 0 – 30% = Not very much for me  31 – 60% = About half for me  61 – 100% = Mostly for me |  |  |  |
|  | - If you decided to reduce the amount you make yourself sick, how confident are you in your ability to do so? (please circle one of the following) | 0 – 30% = Not at all confident  31 – 70 % = Confident  61 – 100% = Extremely confident |  |  |  |
|  | - In the past two weeks, how much of you has wanted to use laxatives to control shape/weight? (please circle one of the following) - In the past two weeks, how much of you has been actively working to reduce your laxative use? (please circle one of the following) | 0 – 30% = A small part of me  31 – 60% = About half of me  61 – 100% = Most of me |  |  |  |
|  | - If you were to reduce your laxative use, how much of this would be for you (versus for others)? (please circle one of the following) | 0 – 30% = Not very much for me  31 – 60% = About half for me  61 – 100% = Mostly for me |  |  |  |
|  | - If you decided to reduce your laxative use, how confident are you in your ability to do so? (please circle one of the following) | 0 – 30% = Not at all confident  31 – 70 % = Confident  61 – 100% = Extremely confident |  |  |  |
|  | - In the past two weeks, how much of you has wanted to use diuretics to control shape/weight? (please circle one of the following) - In the past two weeks, how much of you has been actively working to reduce your diuretic use? (please circle one of the following) | 0 – 30% = A small part of me  31 – 60% = About half of me  61 – 100% = Most of me |  |  |  |
|  | - If you were to reduce your diuretic use, how much of this would be for you (vs. Others)? (circle one of the following) | 0 – 30% = Not very much for me  31 – 60% = About half for me  61 – 100% = Mostly for me |  |  |  |
|  | - If you decided to reduce your diuretic use, how confident are you in your ability to do so? (please circle one of the following) | 0 – 30% = Not at all confident  31 – 70 % = Confident  61 – 100% = Extremely confident |  |  |  |
|  | - In the past two weeks, how much of you has wanted to exercise? (please circle one of the following) - In the past two weeks, how much of you has been actively working to reduce your exercise? (please circle one of the following) | 0 – 30% = A small part of me  31 – 60% = About half of me  61 – 100% = Most of me |  |  |  |
|  | - If you were to reduce your exercise, how much of this would be for you (versus for others)? (please circle one of the following) | 0 – 30% = Not very much for me  31 – 60% = About half for me  61 – 100% = Mostly for me |  |  |  |
|  | - If you decided to reduce your exercise, how confident are you in your ability to do so? (please circle one of the following) | 0 – 30% = Not at all confident  31 – 70 % = Confident  61 – 100% = Extremely confident |  |  |  |
|  | - In the past two weeks, how much of you has wanted to hold on to your fear of weight gain? (please circle one of the following) - In the past two weeks, how much of you has been actively working to reduce your fear of weight gain? (please circle one of the following) | 0 – 30% = A small part of me  31 – 60% = About half of me  61 – 100% = Most of me |  |  |  |
|  | - If you were to reduce your fear of weight gain, how much of this would be for you (versus for others)? (please circle one of the following) | 0 – 30% = Not very much for me  31 – 60% = About half for me  61 – 100% = Mostly for me |  |  |  |
|  | - If you decided to reduce your fear of weight gain, how confident are you in your ability to do so? (please circle one of the following) | 0 – 30% = Not at all confident  31 – 70 % = Confident  61 – 100% = Extremely confident |  |  |  |
|  | - In the past two weeks, how much of you has wanted to hold on to your feelings of fatness? (please circle one of the following) - In the past two weeks, how much of you has been actively working to reduce your feelings of fatness? (please circle one of the following) | 0 – 30% = A small part of me  31 – 60% = About half of me  61 – 100% = Most of me |  |  |  |
|  | - If you were to reduce your feelings of fatness, how much of this would be for you (versus for others)? (please circle one of the following) | 0 – 30% = Not very much for me  31 – 60% = About half for me  61 – 100% = Mostly for me |  |  |  |
|  | - If you decided to reduce your feelings of fatness, how confident are you in your ability to do so? (please circle one of the following) | 0 – 30% = Not at all confident  31 – 70 % = Confident  61 – 100% = Extremely confident |  |  |  |
|  | - In the past two weeks, how much of you has wanted your shape or weight to matter? (please circle one of the following) - In the past two weeks, how much of you has been actively working to make shape or weight matter less? (please circle one of the following) | 0 – 30% = A small part of me  31 – 60% = About half of me  61 – 100% = Most of me |  |  |  |
|  | - If you were to try to make shape or weight matter less, how much of this would be for you (versus for others)? (please circle one of the following) | 0 – 30% = Not very much for me  31 – 60% = About half for me  61 – 100% = Mostly for me |  |  |  |
|  | - If you decided to make shape and weight matter less, how confident are you in your ability to do so? (please circle one of the following) | 0 – 30% = Not at all confident  31 – 70 % = Confident  61 – 100% = Extremely confident |  |  |  |
| ***Current depressive symptomatology*** | Over the last 2 weeks, how often have you been bothered by any of the following problems?   - Little interest or pleasure in doing things - Feeling down, depressed, or hopeless - Trouble falling or staying asleep, or sleeping too much - Feeling tired or having little energy - Poor appetite or overeating - Feeling bad about yourself – or that you are a failure or have let yourself or your family down - Trouble concentrating on things, such as reading the newspaper or watching television - Moving or speaking so slowly that other people could have noticed? Or the opposite – being so fidgety or restless that you have been moving around a lot more than usual - Thoughts that you would be better off dead or of hurting yourself in some way | 0 = Not at all  1 = Several days  2 = More than half the days  3 = Nearly everyday | X | X | Adapted from Kroenke et al., 2001 |
|  | - If you checked off any problems, how difficult have these problems made it for you to do your work, take care of things at home, or get along with other people? | 1. Not difficult at all 2. Somewhat difficult 3. Very difficult 4. Extremely difficult |  |  |  |
| ***Current anxiety symptomatology*** | Over the last 2 weeks, how often have you been bothered by the following problems?   - Feeling nervous, anxious, or on edge - Not being able to stop or control worrying - Worrying too much about different things - Trouble relaxing - Being so restless that it's hard to sit still - Becoming easily annoyed or Irritable - Feeling afraid as if something awful might happen | 0 = Not at all  1 = Several days  2 = More than half the days  3 = Nearly every day | X | X | Adapted from Spitzer et al., 2006 |
|  | If you checked off any problem on this questionnaire so far, how difficult have these problems made it for you to do your work, take care of things at home, or get along with other people? | 1. Not difficult at all 2. Somewhat difficult 3. Very difficult 4. Extremely Difficult |  |  |  |
| ***Body dissatisfaction*** | Over the past four weeks:   - Has feeling bored made you brood about your shape? - Have you been so worried about your shape that you have been feeling you ought to diet? - Have you thought that your thighs, hips, or bottom are too large for the rest of you? - Have you been afraid that you might become fat (or fatter)? - Have you worried about your flesh being not firm enough? - Has feeling full (e.g., after eating a large meal) made you feel fat? - Have you felt so bad about your shape that you have cried? - Have you avoided running because your flesh might wobble? - Has being with thin women made you feel self-conscious about your shape? - Have you worried about your thighs spreading out when sitting down? - Has eating even a small amount of food made you feel fat? - Have you noticed the shape of other women and felt that your own shape compared unfavourably? - Has thinking about your shape interfered with your ability to concentrate (e.g., while watching television, reading, listening to conversations)? - Has being naked, such as when taking a bath, made you feel fat? - Have you avoided wearing clothes which make you particularly aware of the shape of your body? - Have you imagined cutting off fleshy areas of your body? - Has eating sweets, cakes, or other high calorie food made you feel fat? - Have you not gone out to social occasions (e.g., parties) because you have felt bad about your shape? - Have you felt excessively large and rounded? - Have you felt ashamed of your body? - Has worry about your shape made you diet? - Have you felt happiest about your shape when your stomach has been empty (e.g., in the morning)? - Have you thought that you are in the shape you are because you lack self-control? - Have you worried about other people seeing rolls of fat around your waist or stomach? - Have you felt that it is not fair that other women are thinner than you? - Have you vomited in order to feel thinner? - When in company have your worried about taking up too much room (e.g., sitting on a sofa, or a bus seat)? - Have you worried about your flesh being dimply? - Has seeing your reflection (e.g., in a mirror or shop window) made you feel bad about your shape? - Have you pinched areas of your body to see how much fat there is? - Have you avoided situations where people could see your body (e.g., communal changing rooms or swimming baths)? - Have you taken laxatives in order to feel thinner? - Have you been particularly self-conscious about your shape when in the company of other people? - Has worry about your shape made you feel you ought to exercise? | 1 = Never  2 = Rarely  3 = Sometimes  4 = Often  5 = Very Often  6 = Always | X | X | Adapted from Cooper et al., 1987 |
| ***Technology literacy*** | - How useful do you feel the Internet is in helping you in making decisions about your health? - How important is it for you to be able to access health resources on the Internet? | 1 = Not useful at all  2 = Not useful  3 = Unsure  4 = Useful  5 = Very useful | X |  | Adapted from Norman & Skinner, 2006 |
|  | - I know what health resources are available on the Internet - I know where to find helpful health resources on the Internet - I know how to find helpful health resources on the Internet - I know how to use the Internet to answer my questions about health - I know how to use the health information I find on the Internet to help me - I have the skills I need to evaluate the health resources I find on the Internet - I can tell high quality health resources from low quality health resources on the Internet - I feel confident in using information from the Internet to make health decisions | 1. Strongly Disagree 2. Disagree 3. Undecided 4. Agree 5. Strongly Agree |  |  |  |
|  | Using a mobile device I can (basic functions):   - Turn the device on and off - Charge the device when the battery is low - Navigate onscreen menus using the touchscreen - Use the onscreen keyboard to type - Copy and paste text using the touchscreen - Adjust the volume of the device - Adjust the screen brightness - Adjust text size - Connect to a WiFi network | 1 = Never tried  2 = Not at all  3 = Not very easily  4 = Somewhat easily  5 = Very easily | X |  | Adapted from Roque & Boot, 2018 |
|  | Using a mobile device I can (communications):   - Open emails - Send emails - Send the same email to multiple people at the same time - Store email addresses in an email address book or contact list - View pictures sent by email - Send pictures by email - Post messages to Social Media Networks (e.g., Facebook, Twitter, Instagram, Google Plus) - Use instant-messaging (e.g., AIM, Yahoo Messenger, MSN Messenger) - Use video-messaging (e.g., Skype, Google Hangout, FaceTime) | 1 = Never tried  2 = Not at all  3 = Not very easily  4 = Somewhat easily  5 = Very easily |  |  |  |
|  | Using a mobile device I can (data and file storage):   - Transfer information (files such as music, pictures, documents) on my mobile device to my computer - Transfer information (files such as music, pictures, documents) on my computer to my mobile device - Store information with a service that lets me view my files from anywhere (e.g., Dropbox, Google Drive, Microsoft Onedrive) | 1 = Never tried  2 = Not at all  3 = Not very easily  4 = Somewhat easily  5 = Very easily |  |  |  |
|  | Using a mobile device I can (internet):   - Use search engines (e.g., Google, Bing) - Find information about local community resources on the Internet - Find information about my hobbies and interests on the Internet - Find health information on the Internet - Read the news on the Internet - Make purchases on the Internet - Bookmark websites to find them again later (make favorites) - Save text and images I find on the Internet | 1 = Never tried  2 = Not at all  3 = Not very easily  4 = Somewhat easily  5 = Very easily |  |  |  |
|  | Using a mobile device I can (calendar):   - Enter events and appointments into a calendar - Check the date and time of upcoming and prior appointments - Set up alerts to remind me of events and appointments | 1 = Never tried  2 = Not at all  3 = Not very easily  4 = Somewhat easily  5 = Very easily |  |  |  |
|  | Using a mobile device I can (entertainment):   - Use the device’s online “store” to find games and other forms of entertainment (e.g., using Apple App Store or Google Play Store) - Watch movies and videos - Listen to music - Read a book - Take pictures and video | 1 = Never tried  2 = Not at all  3 = Not very easily  4 = Somewhat easily  5 = Very easily |  |  |  |
|  | Using a mobile device I can (privacy):   - Set up a password to lock/ unlock the device - Erase pictures and videos stored on the device - Erase all Internet browsing history and temporary files - Reset the device to factory settings, erasing all account information | 1 = Never tried  2 = Not at all  3 = Not very easily  4 = Somewhat easily  5 = Very easily |  |  |  |
|  | Using a mobile device I can (troubleshooting):   - Restart the device when it is frozen or not working right - Update games and other applications - Close games and other applications - Delete games and other applications - Upgrade device software | 1 = Never tried  2 = Not at all  3 = Not very easily  4 = Somewhat easily  5 = Very easily |  |  |  |
| ***Acceptability and feasibility*** | Perceived Usefulness:   - Using MI-Coach: ED allowed me to access mental health services more quickly. - Using MI-Coach: ED improved my eating disorder symptoms. - Using MI-Coach: ED increased my motivation to recover from my eating disorder. - Using MI-Coach: ED enhanced my ability to stay on my treatment waitlist. - Using MI-Coach: ED made it easier to start my eating disorder recovery. - I found MI-Coach: ED to be useful in my eating disorder recovery. | 1. Extremely likely 2. Quite likely 3. Slightly likely 4. Neither likely nor unlikely 5. Slightly unlikely 6. Quite unlikely 7. Extremely unlikely |  | X | Adapted from Davis, 1989 |
|  | Perceived Ease of Use:   - Learning to use MI-Coach: ED was easy for me. - I found it easy to do what I wanted to do on MI-Coach: ED - Using MI-Coach: ED was clear and understandable - I found MI-Coach: ED to be flexible to interact with. - It was easy for me to become skillful at using MI-Coach: ED. - I found MI-Coach: ED easy to use. | 1. Extremely likely 2. Quite likely 3. Slightly likely 4. Neither likely nor unlikely 5. Slightly unlikely 6. Quite unlikely 7. Extremely unlikely |  |  |  |
|  | Entertainment: Is the app fun/entertaining to use? Does it have components that make it more fun than other similar apps | 1. = Dull, not fun or entertaining at all 2. = Mostly boring 3. = OK, fun enough to entertain user for a brief time (< 5 minutes) 4. = Moderately fun and entertaining, would entertain user for some time (5-10 minutes total) 5. = Highly entertaining and fun, would stimulate repeat use |  | X | Adapted from Stoyanov et al., 2016 |
|  | Interest: Is the app interesting to use? Does it present its information in an interesting way compared to other similar apps? | 1. = Not interesting at all 2. = Mostly uninteresting 3. = OK, neither interesting nor uninteresting; would engage user for a brief time (< 5 minutes) 4. = Moderately interesting; would engage user for some time (5-10 minutes total) 5. = Very interesting, would engage user in repeat use |  |  |  |
|  | Customisation: Does it allow you to customise the settings and preferences that you would like to (e.g., sound, content, and notifications)? | 1. = Does not allow any customisation or requires setting to be input every time 2. = Allows little customisation and that limits app’s functions 3. = Basic customisation to function adequately 4. = Allows numerous options for customisation   Allows complete tailoring the user’s characteristics/preferences, remembers all settings |  |  |  |
|  | Interactivity: Does it allow user input, provide feedback, contain prompts (reminders, sharing options, notifications, etc.)? | 1. = No interactive features and/or no response to user input 2. = Some, but not enough interactive features which limits app’s functions 3. = Basic interactive features to function adequately 4. = Offers a variety of interactive features, feedback and user input options 5. = Very high level of responsiveness through interactive features, feedback and user input options |  |  |  |
|  | Target group: Is the app content (visuals, language, design) appropriate for the target audience? | 1. = Completely inappropriate, unclear or confusing 2. = Mostly inappropriate, unclear or confusing 3. = Acceptable but not specifically designed for the target audience. May be inappropriate/unclear/confusing at times 4. = Designed for the target audience, with minor issues 5. = Designed specifically for the target audience, no issues found |  |  |  |
|  | Performance: How accurately/fast do the app features (functions) and components (buttons/menus) work? | 1. = App is broken; no/insufficient/inaccurate response (e.g. crashes/bugs/broken features, etc.) 2. = Some functions work, but lagging or contains major technical problems 3. = App works overall. Some technical problems need fixing, or is slow at times 4. = Mostly functional with minor/negligible problems 5. = Perfect/timely response; no technical bugs found, or contains a ‘loading time left’ indicator (if relevant) |  |  |  |
|  | Ease of use: How easy is it to learn how to use the app; how clear are the menu labels, icons, and instructions? | 1. = No/limited instructions; menu labels, icons are confusing; complicated 2. = Takes a lot of time or effort 3. = Takes some time or effort 4. = Easy to learn (or has clear instructions) 5. = Able to use app immediately; intuitive; simple (no instructions needed) |  |  |  |
|  | Navigation: Does moving between screens make sense; Does app have all necessary links between screens? | 1. = No logical connection between screens at all /navigation is difficult 2. = Understandable after a lot of time/effort 3. = Understandable after some time/effort 4. = Easy to understand/navigate 5. = Perfectly logical, easy, clear and intuitive screen flow throughout, and/or has shortcuts |  |  |  |
|  | Gestural design: Do taps/swipes/pinches/scrolls make sense? Are they consistent across all components/screens? | 1. = Completely inconsistent/confusing 2. = Often inconsistent/confusing 3. = OK with some inconsistencies/confusing elements 4. = Mostly consistent/intuitive with negligible problems 5. = Perfectly consistent and intuitive |  |  |  |
|  | Layout: Is arrangement and size of buttons, icons, menus, and content on the screen appropriate? | 1. = Very bad design, cluttered, some options impossible to select, locate, see or read 2. = Bad design, random, unclear, some options difficult to select/locate/see/read 3. = Satisfactory, few problems with selecting/locating/seeing/reading items 4. = Mostly clear, able to select/locate/see/read items 5. = Professional, simple, clear, orderly, logically organised |  |  |  |
|  | Graphics: How high is the quality/resolution of graphics used for buttons, icons, menus, and content? | 1. = Graphics appear amateur, very poor visual design - disproportionate, stylistically inconsistent 2. = Low quality/low resolution graphics; low quality visual design – disproportionate 3. = Moderate quality graphics and visual design (generally consistent in style) 4. = High quality/resolution graphics and visual design – mostly proportionate, consistent in style 5. = Very high quality/resolution graphics and visual design - proportionate, consistent in style |  |  |  |
|  | Visual appeal: How good does the app look? | 1. = Ugly, unpleasant to look at, poorly designed, clashing, mismatched colours 2. = Bad – poorly designed, bad use of colour, visually boring 3. = OK – average, neither pleasant, nor unpleasant 4. = Pleasant – seamless graphics – consistent and professionally designed 5. = Beautiful – very attractive, memorable, stands out; use of colour enhances app features/menus |  |  |  |
|  | Quality of information: Is app content correct, well written, and relevant to the goal/topic of the app? | N/A = There is no information within the app   1. = Irrelevant/inappropriate/incoherent/ incorrect 2. = Poor. Barely relevant/appropriate/ coherent/may be incorrect 3. = Moderately relevant/appropriate/ coherent/and appears correct 4. = Relevant/appropriate/coherent/ correct 5. = Highly relevant, appropriate, coherent, and correct |  |  |  |
|  | Quantity of information: Is the information within the app comprehensive but concise? | N/A = There is no information within the app   1. = Minimal or overwhelming 2. = Insufficient or possibly overwhelming 3. = OK but not comprehensive or concise 4. = Offers a broad range of information, has some gaps or unnecessary detail; or has no links to more information and resources 5. = Comprehensive and concise; contains links to more information and resources |  |  |  |
|  | Visual information: Is visual explanation of concepts – through charts/graphs/ images/videos, etc. – clear, logical, correct? | N/A. There is no visual information within the app (e.g., it only contains audio, or text)   1. = Completely unclear/confusing/ wrong or necessary but missing 2. = Mostly unclear/confusing/wrong 3. = OK but often unclear/confusing/ wrong 4. = Mostly clear/logical/correct with negligible issues 5. = Perfectly clear/logical/correct |  |  |  |
|  | Credibility of source: does the information within the app seem to come from a credible source? | N/A = There is no information within the app   1. = Suspicious source 2. = Lacks credibility 3. = Not suspicious but legitimacy of source is unclear 4. = Possibly comes from a legitimate source 5. = Definitely comes from a legitimate/specialised source |  |  |  |
|  | Would you recommend this app to people who might benefit from it? | 1. = Not at all - I would not recommend this app to anyone 2. = There are very few people I would recommend this app to 3. = Maybe - There are several people I would recommend this app to 4. = There are many people I would recommend this app to 5. = Definitely - I would recommend this app to everyone |  |  |  |
|  | How many times do you think you would use this app in the next 12 months if it was relevant to you? | 1. = None 2. = 1-2 3. = 3-10 4. = 10-50 5. = >50 |  |  |  |
|  | Would you pay for this app? | 1. = Definitely not 2. = 3. = 4. = 5. = Definitely yes |  |  |  |
|  | What is your overall (star) rating of the app? | 1. = ★One of the worst apps I’ve used 2. = ★★ 3. = ★★★Average 4. = ★★★★ 5. = ★★★★★ One of the best apps I've used |  |  |  |
|  | - Awareness – This app has increased my awareness of the importance of addressing the health behaviour - Knowledge – This app has increased my knowledge/ understanding of the health behaviour - Attitudes – The app has changed my attitudes toward improving this health behaviour - Intention to change – The app has increased my intentions/motivation to address this health behaviour - Help seeking – This app would encourage me to seek further help to address the health behaviour (if I needed it) - Behaviour change – Use of this app will increase/decrease the health behaviour | 1. = Strongly Disagree 2. = Disagree 3. = Neutral 4. = Agree 5. = Strongly Agree |  |  |  |
|  | Do you have any further comments about the app? | DESCRIPTIVE TEXT |  |  |  |
|  | App use metrics:   - Sessions completed - Exercises completed - Number of days of app use | CLINICIAN BACKEND DATA |  | X | Original |
