# Supplementary material for: Mobile App–Delivered Motivational Interviewing for Women on Eating Disorder Treatment Waitlists (MI-Coach: ED): Protocol for an App Development and Pilot Evaluation
Source: JMIR Res Protoc. 2025 Apr 10;14:e66298. doi: 10.2196/66298 (PMC12022520; doi:10.2196/66298)
Supplement: Multimedia Appendix 5 [file resprot_v14i1e66298_app5.docx]

**BRIEF CONSENT FORM**

**Mobile App-Delivered Motivational Interviewing for Individuals on Eating Disorder Clinic Waitlists: Pilot and Feasibility Study**

Principal Investigators:

Maya Libben, Ph.D., Department of Psychology, UBC

Irving K. Barber School of Arts and Social Sciences, ASC 284

University of British Columbia Okanagan

Email: plan.lab@ubc.ca

Work: 250-807-9026

Co-Investigator:

Amané Halicki-Asakawa, B.A., The PLAN Lab, UBC

**Invitation**

We would like to invite you again to take part of a research study for adult women on waitlists for eating disorder treatment. Given the duration of time that has passed since the initial consent form was signed, the purpose of this form is to revisit whether you would like to continue to take part in the study. This consent form describes the details of the research project, as well as the previous activities to which you consented. Please take time to read the following information carefully and contemplate whether you would like to continue participation. If you *do* wish to participate in this study, you will be asked to sign this form.

**Purpose of the study**

This study will pilot test a mobile-app based intervention, MI-Coach: ED, that is designed to increase motivation to recover from an eating disorder in adult women aged 18 and above. The goal is to determine if the app is an acceptable and feasible way to improve motivation to recover from an eating disorder. This study aims to do this by asking adult women on eating disorder waitlists to use the mobile app for a one-month duration, and to provide their feedback on their experience. The results will be used to improve the mobile app, and to develop future studies that will further evaluate MI-Coach: ED.

**Who is conducting the study?**

The Principal Investigator, Dr. Maya Libben, and the Co-Investigator, Amané Halicki-Asakawa, are researchers from the University of British Columbia in the Okanagan. This study is part of Amané Halicki-Asakawa’s Master’s thesis, which is a public document that will be available on the Internet through UBC cIRcle.

**What did I consent to previously?**

During the previous consent process, you agreed to take part in the following study components (estimated to take 5-14 hours in total): the completion of two sets of questionnaires (taking up to 1 hour each, online), an orientation to the MI-Coach: ED app (taking approximately 45 minutes), use of the app month for a 1-month duration, and a 1-hour recorded Zoom interview asking about your experiences using the app. This consent form revisits whether you would like to continue with the completion of the second set of questionnaires and the Zoom interview and will take approximately 2 hours in total.

**Your participation is voluntary**

Your participation in this study is entirely voluntary. If you do not wish to take part, there will be no disadvantage of any kind to you and we thank you for considering our request.

**What are the possible harms and discomforts?**

If you participate in this study, there are risks no greater than what you would experience in your daily life. Some of the questions or topics in the study may make you uncomfortable. You may choose not to answer those questions.

You may also withdraw from this study by closing your browser to exit the survey or leaving the Zoom meeting. If you choose to withdraw, all of your data, including from the first portion of the study, will be deleted and omitted from analysis.

**What are the potential benefits of participating?**

This research study will provide important scientific information and our findings can help find ways to improve service access for women with eating disorders in the future.

**Will I be paid for participating in the study?**

You will be offered $10 CAD for each study component completed (i.e., orientation, two sets of questionnaires, interview), resulting in a total of $40 CAD compensation via online gift card for full participation. In addition, you will be provided with 1-year access to the MI-Coach © app (valued at $122 CAD).

**How will my taking part in this study be kept confidential?**

Your confidentiality will be respected. All information collected from this study will be kept completely confidential. All documents will be identified only by code number and stored electronically. You will not be identified by name in any reports or presentations of the completed study.

The online questionnaires will be administered by the UBC-hosted version of Qualtrics. All data will be stored and backed up in Canada. Once the data has been downloaded to a confidential and password-protected laboratory computer, they will be permanently deleted from the Qualtrics servers.

Zoom interviews will be conducted using a UBC licensed version of Zoom, which uses servers located in Canada. The Zoom interview will be recorded and then transcribed, after which the recording will be permanently deleted. All identifying information will be deleted from the interview transcripts.

All data entered into the mobile app will be protected and kept confidential according to Resiliens Inc.’s privacy policy (<https://resiliens.com/privacy>), the developers of the MI-Coach: ED app. All data security practices are in line with the US’ Health Insurance Portability and Accountability Act (“HIPAA”). Resiliens Inc.’s data is stored through the Amazon Web Services platform on secure servers located in North Virginia, USA, and will be permanently deleted once the data is securely transferred to project researchers.

The data on which the results of the project depend upon will be retained in secure storage for 5 years after the research has been published. After this time, they will be destroyed.

Your rights to privacy are legally protected by federal and provincial laws that require safeguards to ensure that your privacy is respected. Further details about these laws are available on request to your study Investigator.

**Who do I contact if I have questions about the study during my participation?**

Please feel free to contact us at any time with questions and concerns you may have about participating in this research study. You are also welcome to contact Dr. Libben to request the results of the study.

**Who do I contact if I have any questions or concerns about my rights as a participant?**

If you have any concerns or complaints about your rights as a research participant and/or your experiences while participating in this study, contact the Research Participant Complaint Line in the University of British Columbia Office of Research Ethics by e-mail at RSIL@ors.ubc.ca or by phone at 604-822-8598 (Toll Free: 1-877-822-8598). Please reference the study number H22-02046 when calling so the Complaint Line staff can better assist you.

**BRIEF CONSENT FORM**

**Mobile App-Delivered Motivational Interviewing for Individuals on Eating Disorder Clinic Waitlists: Pilot and Feasibility Study**

Participant Consent

**Signatures**

By signing the section below, you acknowledge that all of your questions pertaining to the current study have been answered, and consent to participate is given. Your participation in this study is entirely voluntary and you may withdraw from the study at any time.

# I consent to participate in this study.

# I consent to the recording of the Zoom assessment.

#

My Name

_________________ ____

Signature Date
