# Supplementary material for: Mobile App–Delivered Motivational Interviewing for Women on Eating Disorder Treatment Waitlists (MI-Coach: ED): Protocol for an App Development and Pilot Evaluation
Source: JMIR Res Protoc. 2025 Apr 10;14:e66298. doi: 10.2196/66298 (PMC12022520; doi:10.2196/66298)
Supplement: Multimedia Appendix 6 [file resprot_v14i1e66298_app6.docx]

**General Questions**

1. How are you doing today?
   1. *Follow up:* How are you doing in regard to your eating disorder recovery?
2. What has your experience been so far in terms of being on a waitlist for eating disorder treatment?
   1. *Follow up:* How many treatment sites have you been waitlisted for?
   2. *Follow up:* How long have you been waiting for services?
   3. *Follow up:* Have you been able to access any services in the interim (e.g., self-help books, websites, peer support groups, etc.)
3. What experience do you have in regard to internet- and mobile-app based mental health services, aside from MI-Coach: ED?
   1. *If applicable:* Of those that you have accessed, how many have been specific to eating disorder recovery?

**Expectations**

1. Recall your experience prior to starting the program: what were your expectations and hopes for MI-Coach: ED?
   1. *Follow up:* How did you think this mobile app would work for you?
   2. *Follow up:* Was there anything that you looked forward to? That you didn’t look forward to?
2. What has or has not met your expectations?
3. How has your view of the program changed since you started using MI-Coach: ED?
4. How has your experience been different from previous interventions you accessed for your eating disorder (e.g., face-to-face treatment, self-help programs, online, etc.)?

**Acceptability**

1. What were your first thoughts when you first started using MI-Coach: ED?
2. How often did you find yourself using the mobile app on a daily/weekly basis?
   1. *Follow up:* How many sessions did you get through?
   2. *Follow up:* How many exercises did you complete?
3. What was your favourite part of the app? What was your least-favourite part?

**Content**

1. What changes (for the better/the worse), if any, have you noticed in yourself through using the MI-Coach: ED app (e.g., eating disorder behaviours, motivation to recover, outlook on staying on treatment waitlists, feelings about future, relationship with others)?
2. How has the mobile-app helped/not helped you prepare for treatment?
3. Which aspect of the MI-Coach: ED app was the most/least helpful to you?
   1. Video sessions
   2. Exercises
   3. Articles
4. Which session of MI-Coach: ED did you find most helpful?
5. Is there any aspect of the mobile app that you feel should be changed?
   1. *Follow up:* Is there anything that you feel was missed?

**Barriers/Facilitators**

1. What was easy/difficult for you in trying to integrate MI-Coach: ED into your daily routine?
   1. *Follow up:* Any technology-related factors? (e.g., interface, notifications, user experience)
   2. *Follow up:* Any personal factors?
2. Is there any other way that you feel that MI-Coach: ED can be improved? (e.g., how it looks, session content, layout, etc.)
3. Is there anything else about your experience with MI-Coach: ED that you wished to share?
